# Association of semen leukocytes with sperm DNA fragmentation in a clinical cohort

**DOI:** 10.3389/fendo.2026.1832477

**Published:** 2026-05-21

**Authors:** Lijun Peng, Tengfei Wang, Mengyi Zhu, Yun Le, Sheng Yang

**Affiliations:** Department of Assisted Reproduction, Huzhou Maternity & Child Health Care Hospital, Huzhou, Zhejiang, China

**Keywords:** albumin, calibration, CASA kinematics, inflammation, logistic regression, male infertility, nonlinear exposure response, predictive model

## Abstract

**Background:**

Sperm DNA fragmentation index (DFI) is increasingly used to characterize male reproductive health, yet its relationships with routinely measured semen parameters, computer-assisted semen analysis (CASA) kinematics, and systemic biomarkers remain incompletely described in clinical practice.

**Methods:**

A total of 1679 semen examination records were included in the analysis. Correlations between DFI and various biomarkers were assessed using Spearman rank correlations. Nonlinear associations were modeled using natural cubic splines, and predictive modeling was performed using multivariable logistic regression.

**Results:**

DFI was right-skewed and increased with age, while differences across abstinence duration strata were modest. Conventional semen parameters and CASA kinematics were inversely correlated with DFI, including progressive motility (PR) and multiple motion descriptors. Semen leukocytes correlated positively with DFI and showed a nonlinear pattern, while albumin exhibited the strongest inverse systemic signal. In prediction analyses, the model showed good discrimination with an area under the curve (AUC) of 0.783, and favorable calibration with a Brier score of 0.080 and expected calibration error (ECE) of 0.015. The association for semen leukocytes remained consistent across alternative high DFI thresholds.

**Conclusions:**

DFI increased with age and was inversely related to semen motility and CASA kinematics in routine clinical practice. Semen leukocytes and albumin were consistent biomarkers associated with sperm DNA fragmentation, with evidence of nonlinear exposure-response patterns. An exploratory multivariable model showed good internal cross-validation performance for identifying high DFI. Further external validation is required before clinical application.

## 1 Introduction

Male factor infertility represents a significant component of global subfertility, and conventional semen analysis remains the primary diagnostic tool in clinical practice. However, basic semen parameters often exhibit limited sensitivity in reflecting sperm genomic integrity and functional competence. The World Health Organization (WHO) laboratory manual emphasizes standardized protocols while advocating for supplemental assessments, such as sperm DNA integrity testing, to refine the diagnostic evaluation (1, 2). Among these, the sperm DFI has emerged as a critical biomarker, as it quantifies cumulative genomic damage with profound implications for fertilization, embryonic development, pregnancy maintenance, and offspring health (3–6).

Sperm DNA fragmentation encompasses a spectrum of defects, including single- and double-strand breaks, oxidative base modifications, and impaired chromatin packaging. Currently, various methodologies are employed to quantify DFI-most notably the sperm chromatin structure assay, TUNEL, comet assays, and sperm chromatin dispersion—each characterized by distinct technical specifications and interpretive nuances (7–10). Clinical consensus supports the utility of DFI assessment in specific contexts, such as unexplained infertility, recurrent pregnancy loss, or varicocele (11). Evidence indicates that elevated DFI levels are associated with diminished clinical pregnancy rates and increased incidence of pregnancy loss, although reported effect sizes vary across different assays and treatment modalities (12, 13).

Oxidative stress constitutes a central mechanistic link between DNA fragmentation and impaired reproductive outcomes. Excessive reactive oxygen species (ROS) can induce lipid peroxidation and protein oxidation, thereby compromising paternal genomic stability (14–17). Inflammatory processes, particularly the presence of semen leukocytes, exacerbate oxidative stress and contribute to reproductive dysfunction (18, 19). Semen leukocytes serve as accessible indicators of this local inflammatory activity, potentially impairing sperm function through localized cytokine signaling and ROS generation (20, 21). Recent reviews on leukocytospermia further emphasize its multifactorial etiologies and impact on DNA integrity (22). Additionally, systemic biomarkers such as serum albumin may reflect the broader antioxidant and nutritional status, yet their association with DFI remains insufficiently explored within a unified analytical framework (23).

Furthermore, DFI appears intertwined with sperm kinematic patterns quantified by CASA. Contemporary CASA systems incorporate modules for DNA integrity and morphology, enhancing the interpretability of semen phenotypes when integrated with DFI data (24–26). Specific CASA kinematics have been shown to correlate with DFI and may improve the diagnostic value of motion descriptors (27). Advanced paternal age is also a recognized determinant; age-related physiological shifts in chromatin repair capacity frequently lead to increased fragmentation, although these patterns exhibit variability across different populations (28–30).

Despite these advancements, comprehensive evaluations integrating systemic biomarkers, conventional parameters, CASA kinematics, and semen leukocytes remain scarce. The present study, therefore, aims to characterize the distribution of DFI and evaluate its multifaceted correlations across systemic and semen-specific domains. We further sought to model potential nonlinear exposure–response relationships and develop an internally validated framework for high DFI risk assessment. By integrating multi-domain biomarkers, this work intends to enhance the clinical interpretability of DFI and identify accessible markers to support routine reproductive evaluation.

## 2 Materials and methods

### 2.1 Study design and data source

This retrospective observational study was based on collected clinical data from the Reproductive Center of Huzhou Maternal and Child Health Care Hospital (Huzhou, China). The study period extended from 3 January 2022 to 31 December 2024 and comprised consecutive semen examinations performed as part of routine clinical care, with concurrent assessment of DFI when available. The analytic dataset was extracted from electronic laboratory and clinical records and curated in a structured spreadsheet format. All analyses in the present study were performed using prespecified variables from the dataset. No additional experiments or external data sources were used.

### 2.2 Participants and measurements

Because this study was based on routinely collected clinical data, the source population reflected consecutive clinical examinations rather than a prospectively recruited research cohort. Semen examination records were eligible if a DFI value was available from the same clinical encounter. The analytic unit was the semen examination record. Accordingly, the same individual could contribute more than one eligible examination during the study period, and the present analyses were conducted at the examination-record level rather than as a longitudinal participant-level analysis.

Semen samples were collected according to the routine protocol of our reproductive center after 2–7 days of abstinence. Routine semen examination included assessment of liquefaction, semen volume, semen pH, sperm concentration, motility, and related parameters, together with CASA-based kinematic evaluation. DFI, semen leukocytes, and other sperm functional indicators were assessed within the routine andrology laboratory workflow using flow cytometry-based fluorescence assays according to the standard protocols applied in our center.

Demographic and clinical information recorded at the time of testing included age and abstinence duration. Abstinence duration was converted to days for analysis. Laboratory variables reflecting systemic inflammation and metabolic status were obtained from routine hospital laboratory records, including platelet count, neutrophil count, lymphocyte count, monocyte count, albumin, fasting glucose, triglycerides, and high-sensitivity C-reactive protein. These biomarkers were measured as part of routine hospital laboratory testing using conventional clinical laboratory methods. Derived indices were calculated when component variables were present, including neutrophil-to-lymphocyte ratio(NLR), platelet-to-lymphocyte ratio(PLR), and systemic immune-inflammation index(SII).

### 2.3 Outcomes and covariates

The primary outcome DFI, analyzed as a continuous variable. For secondary analyses, high sperm DNA fragmentation was defined *a priori* as DFI at least 30 in the main analysis. Alternative thresholds for high DFI were evaluated in robustness analyses.

All association models used a prespecified covariate adjustment set selected *a priori* based on clinical relevance and potential confounding. The adjustment set included age, abstinence days, semen volume, and semen pH.

### 2.4 Statistical analysis

Continuous variables were summarized as mean with standard deviation or median with interquartile range. The distribution of DFI was characterized using histograms with density overlays and empirical cumulative distribution functions. DFI distributions and the prevalence of high DFI were additionally presented across strata of age and abstinence duration. Pairwise associations among semen parameters, CASA kinematics, and available systemic biomarkers were evaluated using Spearman rank correlation coefficients. Pairwise complete-case sample sizes were reported for each correlation. For correlations involving DFI, associations were ranked by the absolute value of the correlation coefficient. Multiple comparisons were addressed by controlling the false discovery rate using the Benjamini-Hochberg procedure.

Associations with DFI as a continuous outcome were examined using multivariable linear regression. Candidate biomarkers for adjusted regression included neutrophil count, lymphocyte count, monocyte count, platelet count, albumin, fasting glucose, triglycerides, semen leukocytes, neutrophil-to-lymphocyte ratio(NLR), and systemic immune-inflammation index(SII). Biomarker and covariate values required for these models were median-imputed, and biomarkers were standardized within the analytic dataset so that coefficients could be interpreted per 1 standard deviation increase. Two nested models were presented: Model 1 adjusted for age and abstinence days, and Model 2 additionally adjusted for semen volume and semen pH.

Potential nonlinearity was evaluated using natural cubic spline models. For each biomarker carried forward to nonlinear modeling, the exposure was represented using a natural cubic spline basis with four knots placed at the empirical 5th, 35th, 65th, and 95th percentiles of the observed exposure distribution, corresponding to 3 spline basis terms in the implemented model. Nonlinear models were fit for a prespecified set of clinically relevant biomarkers with adequate data availability and interpretability, namely albumin, semen leukocytes, neutrophil count, lymphocyte count, platelet count, NLR, and SII. Spline models were adjusted for age, abstinence days, semen volume, and semen pH and were fit on complete-case records for the corresponding exposure-covariate set. For display, adjusted exposure-response curves and 95% confidence intervals were generated with covariates held at their median values, and local slopes were obtained from the fitted curve.

Prediction of high DFI was performed using multivariable logistic regression, with high DFI defined *a priori* as DFI≥30. Predictors with>60% missingness in the analytic dataset were excluded before model fitting. The final predictive model included 15 predictors: age, abstinence days, semen volume, semen pH, total sperm number, sperm concentration, progressive motility(PR), non-progressive motility(PRNP), curvilinear velocity(VCL), straight-line velocity(VSL), average path velocity(VAP), straightness(STR), beat cross frequency(BCF), amplitude of lateral head displacement(ALH), and semen leukocytes. Internal validation was implemented using 5-fold stratified cross-validation at the examination-record level with shuffling and a fixed random state of 2026. Within each training fold, missing predictor values were imputed using the median and predictors were standardized using a pipeline; the fitted model was then applied to the corresponding validation fold to obtain out-of-fold predicted probabilities. Pooled out-of-fold predictions were used to estimate the receiver ROC, AUC, Brier score, and ECE. Calibration was summarized using quantile-based bins (10 bins), and a 95% confidence interval for the AUC was obtained by bootstrap resampling of the pooled out-of-fold predictions.

Robustness was assessed by repeating adjusted logistic regression analyses for semen leukocytes across alternative high-DFI thresholds of 20, 25, 30, and 35, using complete-case data within each threshold-specific model and reporting odds ratios per 1 standard deviation increase adjusted for age, abstinence days, semen volume, and semen pH. Because the analyses were conducted at the semen examination-record level, the reported associations and predictive performance estimates should be interpreted at the level of clinical examination records rather than as within-individual longitudinal effects.

### 2.5 Missing data and quality control

Data completeness differed across variable domains. Semen parameters and CASA kinematics were available for most examination records, whereas systemic inflammatory and metabolic biomarkers were available for a smaller subset. Analyses were therefore conducted using prespecified missing data strategies. Correlation analyses used pairwise complete observations, and the corresponding pairwise sample sizes were reported. Regression analyses reported model specific effective sample sizes according to the variables required for each model. For prediction modeling, missing predictor values were handled using median imputation within the modeling pipeline.

Quality control procedures were applied prior to analysis. Variables were harmonized across records, converted to numeric format where appropriate, and screened for implausible values. Derived inflammatory indices were computed only when component variables were available.

## 3 Results

### 3.1 Study population and analytic sample

The analytic cohort included 1679 semen examination records with available DFI measurements. the median abstinence duration was 3 (3–5) days, the median semen volume was 3.2 (2.3-4.2) mL, and the median semen pH was 7.4 (7.4-7.4).

Using the prespecified main definition of high DFI (≥30%), 183 participants met criteria for high DFI, corresponding to a prevalence of 10.9%. Data completeness differed across variable domains. Semen parameters and CASA kinematics were available for most participants, supporting stable estimation for semen centered analyses. In contrast, systemic inflammatory and metabolic biomarkers were available for a smaller subset of participants. Therefore, effective sample sizes varied across analyses. Correlation analyses were conducted using pairwise complete observations with the corresponding pairwise sample sizes reported. Regression and prediction analyses followed the prespecified modeling strategy described in Methods, with model specific sample sizes reported accordingly.

### 3.2 Distribution of sperm DNA fragmentation and prevalence of high DNA fragmentation

**Figure 1:**
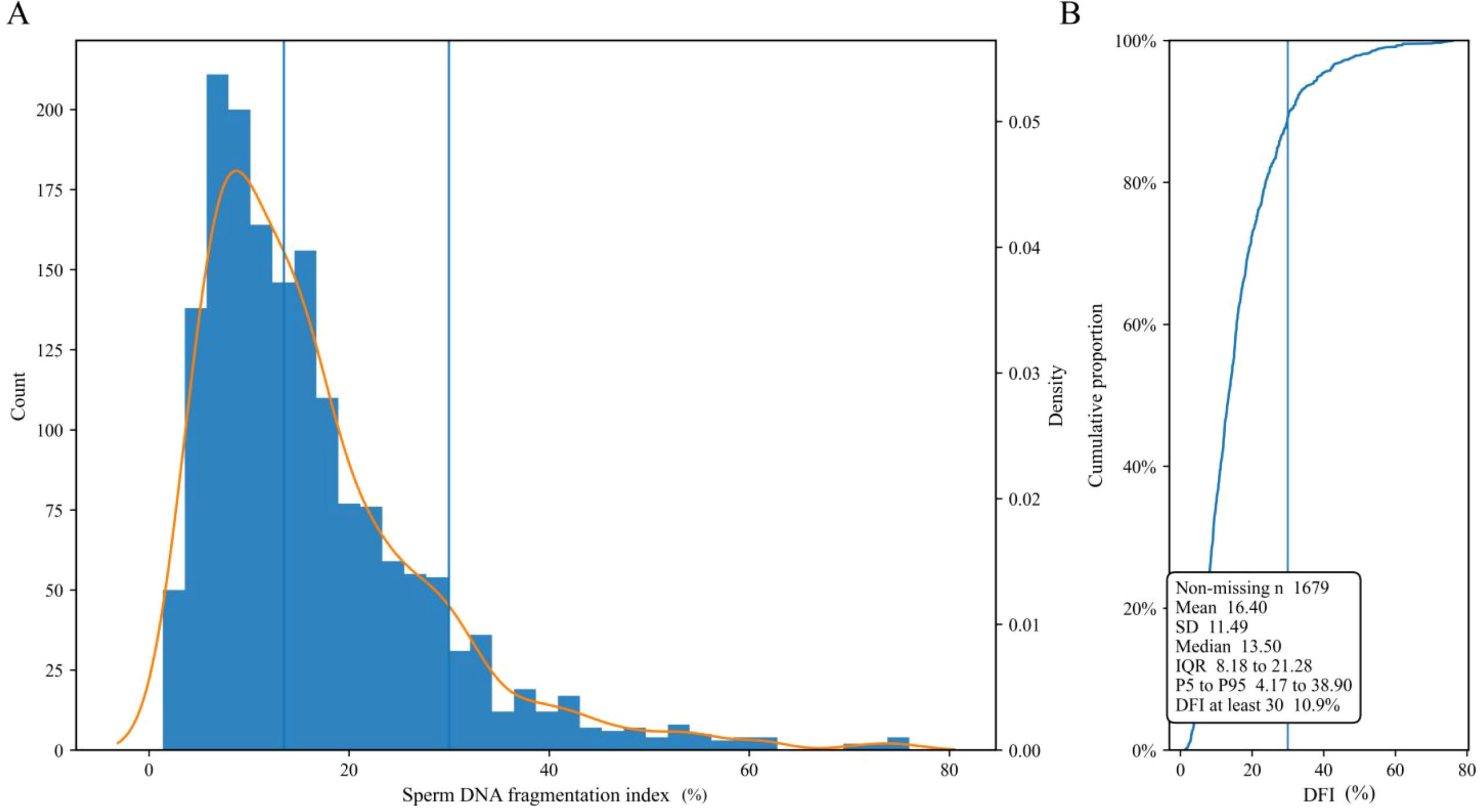
Distribution of DFI in the analytic cohort. The image summarizes the overall distribution of DFI in 1679 examinations. **(A)** shows the histogram of DFI values with an overlaid density curve. **(B)** shows the empirical cumulative distribution of DFI. DFI, sperm DNA fragmentation index.

DFI displayed a right skewed distribution with marked interindividual variability, as shown in **Figure 1**. In the analytic cohort of 1679 examinations, the mean DFI was 14.60 with a standard deviation of 11.49. The median DFI was 13.50% with an interquartile range of 8.18 to 21.28. The 5th percentile was 4.17 and the 95th percentile was 38.90, consistent with a long right tail. Using the prespecified main definition of high DNA fragmentation, defined as DFI at least 30, 183 examinations met criteria for high DFI, yielding an overall prevalence of 10.9%. The empirical cumulative distribution further indicated that a substantial fraction of examinations clustered at relatively low DFI values while a smaller subset contributed disproportionately to the upper tail, supporting the use of both continuous DFI and a threshold based high DFI definition in subsequent analyses.

**Figure 2:**
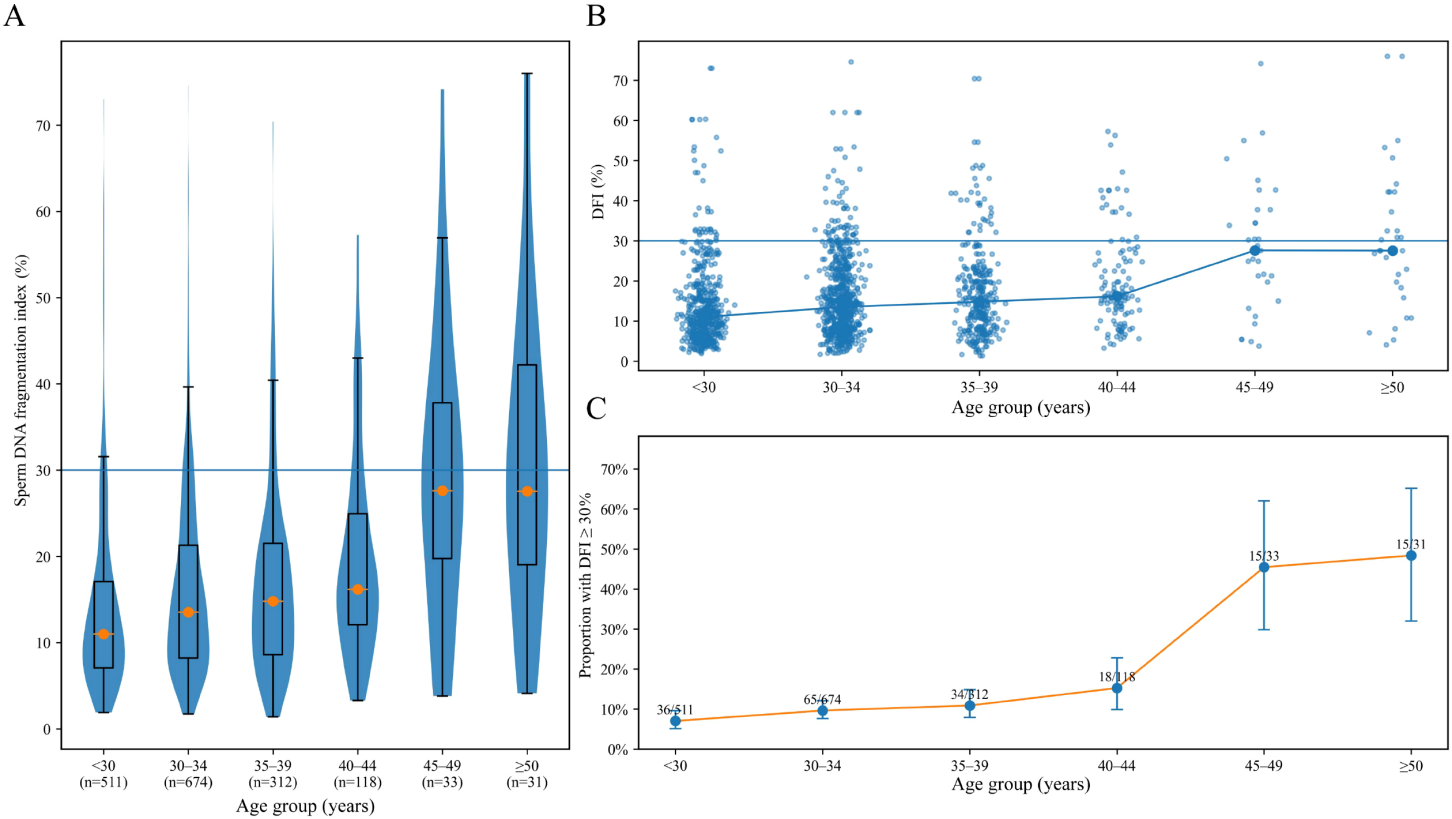
Distribution of DFI across age groups. The image shows DFI distributions across predefined age strata (<30, 30–34, 35–39, 40–44, 45–49, and ≥50 years). **(A)** Violin and box plots showing the distribution of DFI across age groups. **(B)** Scatter plot showing DFI values across age groups with a fitted trend line. **(C)** Line and error bar plot showing the prevalence of high DFI, defined as DFI ≥30%, across age groups. DFI, sperm DNA fragmentation index.

DFI differed across age strata, as shown in **Figure 2**. The distribution shifted upward with increasing age and exhibited greater dispersion in older strata, with a more prominent upper tail. The prevalence of high DFI increased across age groups. It was 7.0% among those younger than 30 years, 9.6% among those aged 30 to 34 years, 10.9% among those aged 35 to 39 years, and 15.3% among those aged 40 to 44 years. In the oldest strata, high DFI prevalence was substantially higher, reaching 45.5% among those aged 45 to 49 years and 48.4% among those aged 50 years or older. These estimates were based on smaller sample sizes, but the Wilson interval plots indicated that the direction of the association was consistent. At the individual level, the scatter plots showed wide within group variability across all ages, indicating that age explains part but not all of the heterogeneity in DFI.

**Figure 3:**
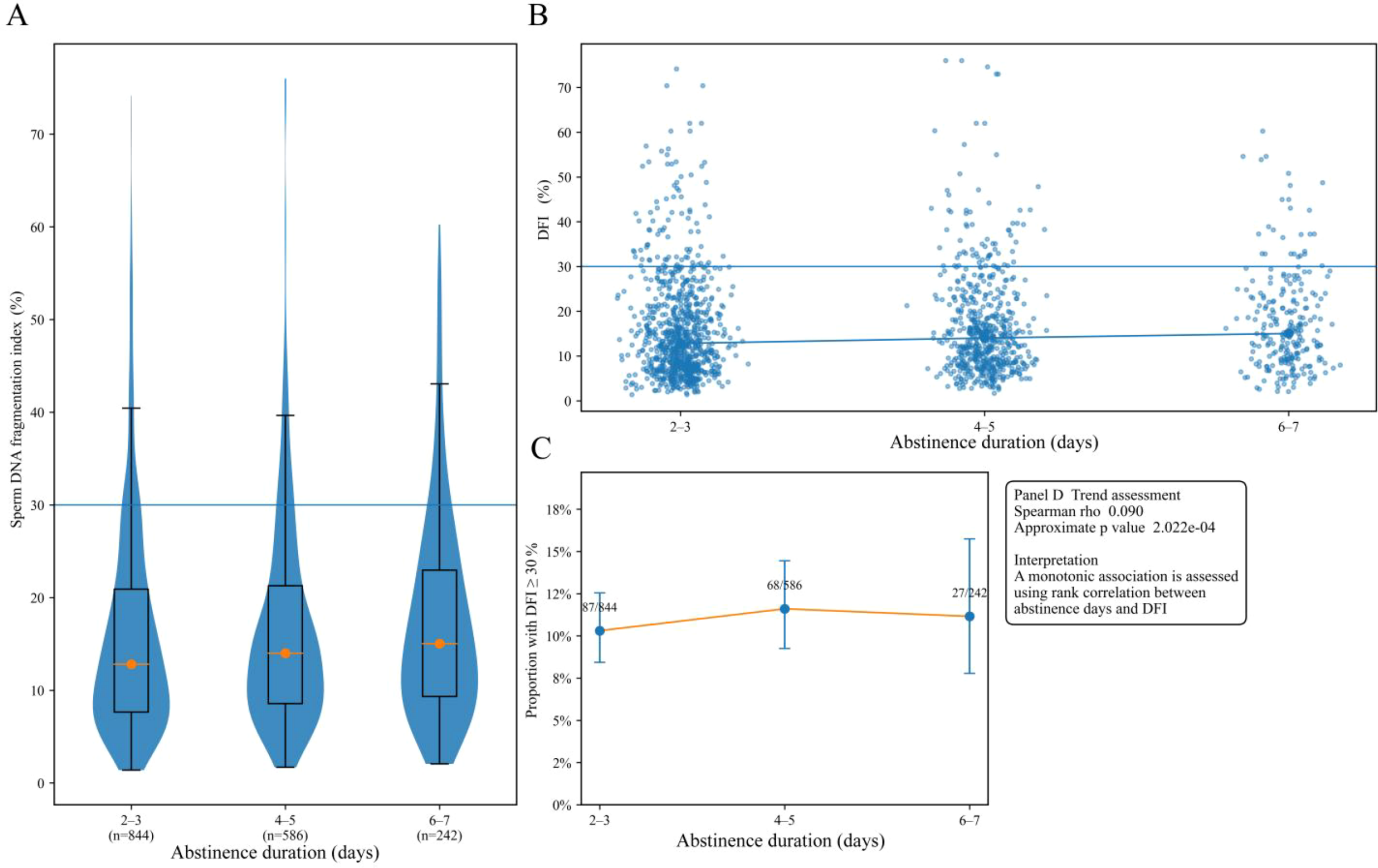
Distribution of DFI across abstinence duration groups. The image shows DFI distributions across abstinence duration groups (2–3 days, 4–5 days, and 6–7 days). **(A)** Violin and box plots showing the distribution of DFI across abstinence duration groups. **(B)** Scatter plot showing DFI values across abstinence duration groups with a fitted trend line. **(C)** Line and error bar plot showing the prevalence of high DFI, defined as DFI ≥30%, across abstinence duration groups, with an inset showing the trend assessment. DFI, sperm DNA fragmentation index.

DFI showed comparatively modest differences across abstinence duration strata, as shown in **Figure 3**. The central tendency was similar across groups, and group level differences were more evident in the upper tail than in the median. The prevalence of high DFI was 10.3% in the 2 to 3 day group, 11.6% in the 4 to 5 day group, and 11.2% in the 6 to 7 day group. A trend assessment indicated a small monotonic association between abstinence days and DFI. Taken together, these results suggest that age related shifts in DFI and high DFI prevalence are more pronounced than differences across the examined abstinence duration groups, while substantial residual heterogeneity remains for subsequent biomarker and semen phenotype analyses.

### 3.3 Correlations of inflammatory and metabolic markers with DFI

Correlations of inflammatory and metabolic markers with DFI are presented in **Table 1**. In this domain, most associations were weak. Albumin showed the strongest inverse correlation with DFI and was the only marker that remained significant after false discovery rate correction. The remaining markers, including fasting glucose, triglycerides, PLR, monocyte count, and platelet count, showed limited associations and did not remain significant after correction for multiple testing. Given the smaller effective sample size for several blood biomarkers, these results should be interpreted cautiously.

**Table 1:** Significance annotations are based on two-sided P values: *P < 0.05 and ***P < 0.001.

Variable	Spearman’s r	n	P value	FDR-adjusted q value	Significance
Albumin	-0.205	418	<0.001	<0.001	***
Fasting glucose	0.186	77	0.105	0.259	
Triglycerides	-0.160	62	0.214	0.306	
PLR	-0.111	394	0.028	0.141	*
Monocyte count	0.080	394	0.115	0.259	
Platelet count	-0.076	394	0.130	0.259	

Data are Spearman rank correlations with DFI. P values are two-sided. q values were adjusted using the Benjamini-Hochberg false discovery rate method. Significance annotations are based on two-sided P values: *P < 0.05, **P < 0.01, ***P < 0.001.

### 3.4 Correlations of semen parameters and CASA kinematic parameters with DFI

Correlations of conventional semen parameters and CASA kinematic parameters with DFI are presented in **Tables 2** and **3**, respectively. Compared with systemic biomarkers, semen-related variables showed clearer associations with DFI.

**Table 2:** Correlations of conventional semen parameters with DFI.

Variable	Spearman’s r	n	P value	FDR-adjusted q value	Significance
PR	-0.390	1679	<0.001	<0.001	***
Total motility	-0.370	1679	<0.001	<0.001	***
Normal morphology	-0.249	222	<0.001	<0.001	***
Semen leukocytes	0.210	1033	<0.001	<0.001	***
Sperm concentration	-0.189	1679	<0.001	<0.001	***
Total sperm number	-0.137	1679	<0.001	<0.001	***

Data are Spearman rank correlations with DFI. P values are two-sided. q values were adjusted using the Benjamini-Hochberg false discovery rate method. Significance annotations are based on two-sided P values: *P < 0.05, **P < 0.01, ***P < 0.001.

**Table 3:** Correlations of CASA kinematic parameters with DFI.

Variable	Spearman’s r	n	P value	FDR-adjusted q value	Significance
VAP	-0.369	1679	<0.001	<0.001	***
BCF	-0.357	1679	<0.001	<0.001	***
VCL	-0.357	1679	<0.001	<0.001	***
VSL	-0.352	1679	<0.001	<0.001	***
STR	-0.350	1679	<0.001	<0.001	***
ALH	-0.341	1679	<0.001	<0.001	***

Data are Spearman rank correlations with DFI. P values are two-sided. q values were adjusted using the Benjamini-Hochberg false discovery rate method. Significance annotations are based on two-sided P values: *P < 0.05, **P < 0.01, ***P < 0.001.

In the conventional semen domain, PR, total motility, normal morphology, sperm concentration, and total sperm number were inversely correlated with DFI, whereas semen leukocytes were positively correlated with DFI. In the CASA domain, VAP, BCF, VCL, VSL, STR, and amplitude of ALH all showed moderate inverse correlations with DFI. Together, these findings indicate that higher DFI was associated with poorer semen quality and less favorable sperm motion characteristics.

### 3.5 Nonlinear exposure response relationships for key biomarkers

**Figure 4:**
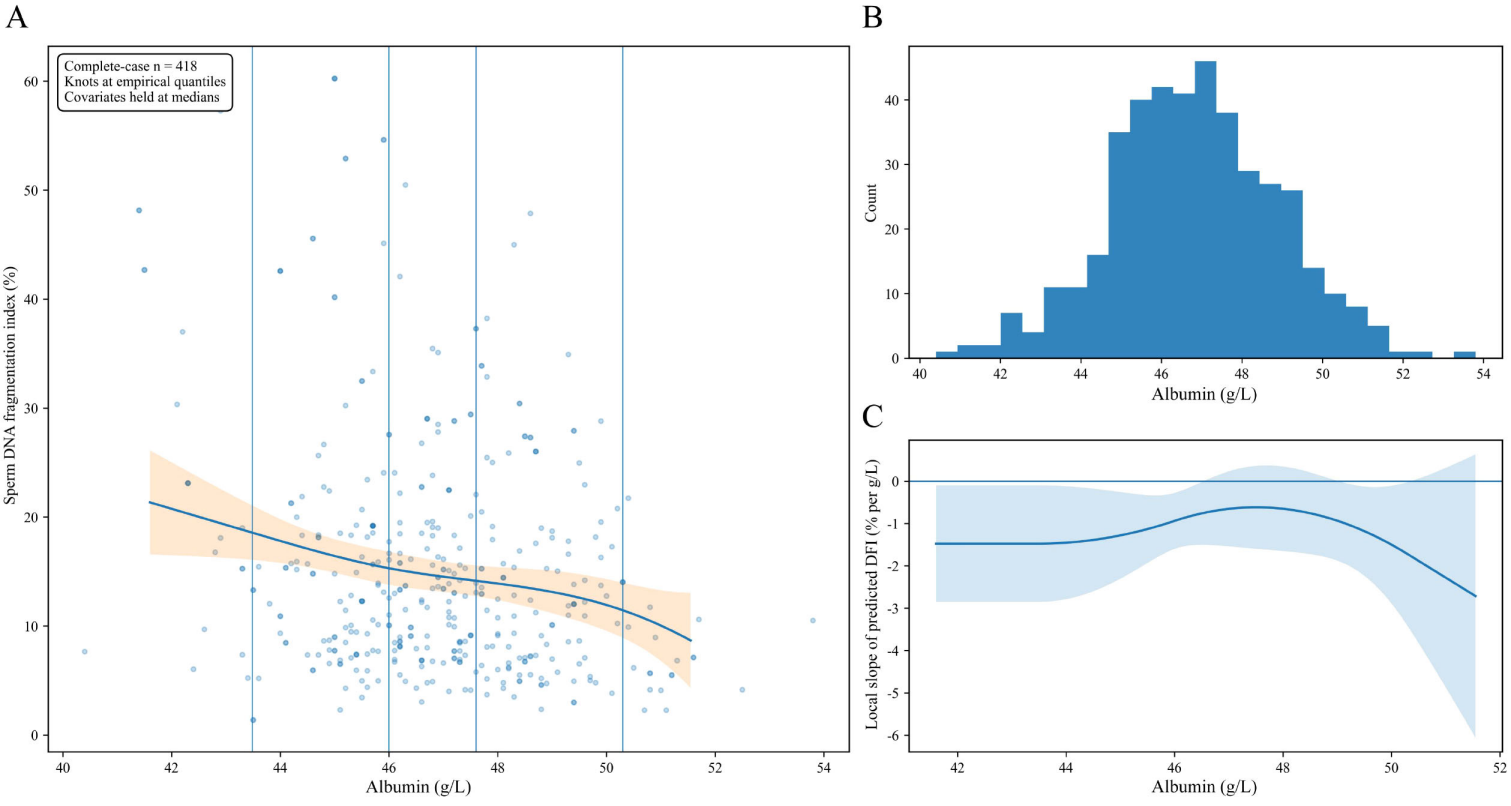
Natural cubic spline models showing the nonlinear association between albumin and DFI. The Y-axis in panel **(A)** represents the predicted mean DFI and its 95% confidence interval. The X-axis shows albumin level. **(A)** shows the adjusted spline curve for the association between albumin and DFI. **(B)** shows the distribution of albumin values in the analytic sample. **(C)** shows the local slope of the fitted spline curve across the observed albumin range. The models were adjusted for age, abstinence days, semen volume, and semen pH. DFI, sperm DNA fragmentation index.

**Figure 5:**
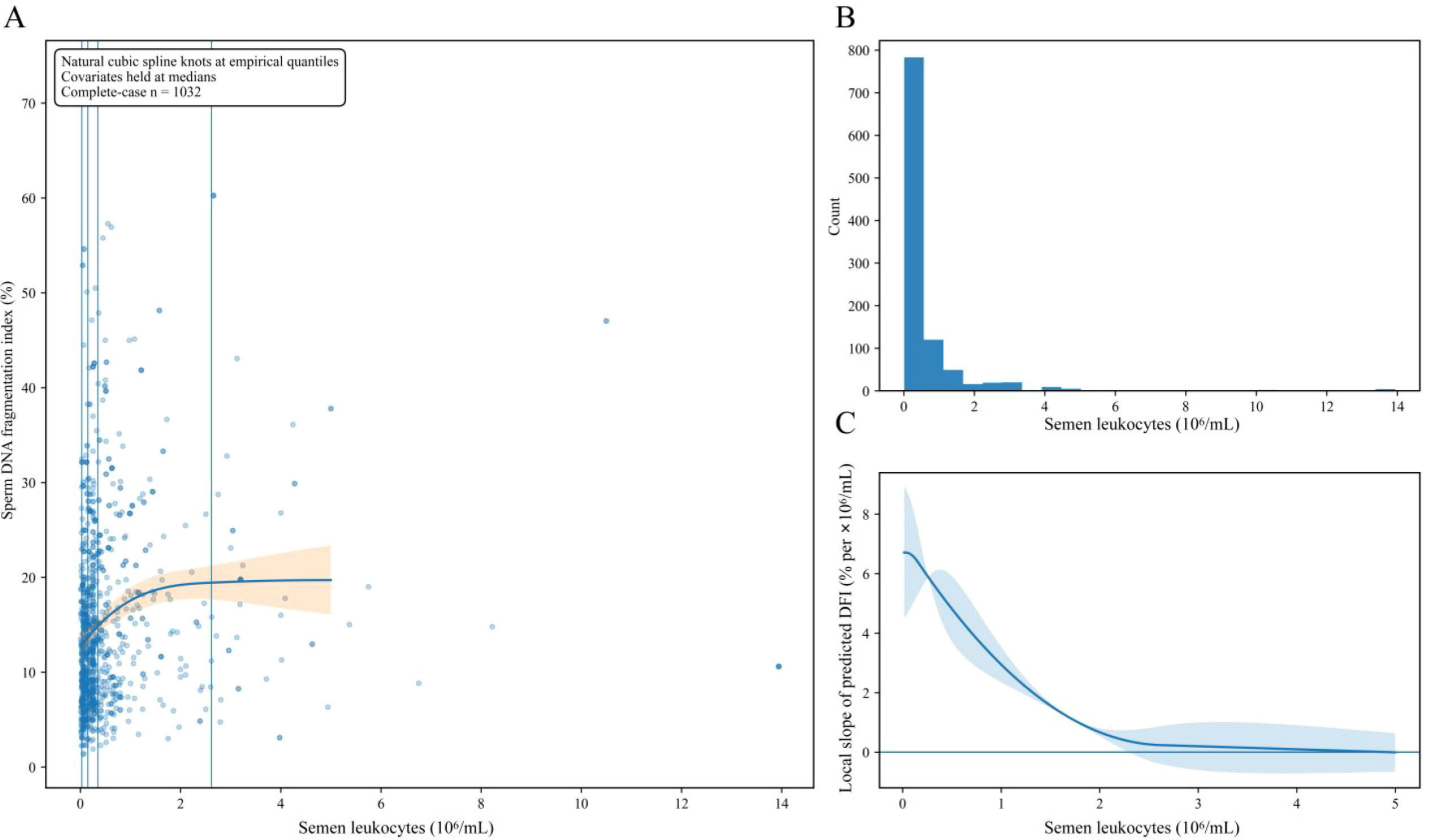
Natural cubic spline models showing the nonlinear association between semen leukocytes and DFI. The Y-axis in panel **(A)** represents the predicted mean DFI and its 95% confidence interval. The X-axis shows semen leukocyte level. **(A)** shows the adjusted spline curve for the association between semen leukocytes and DFI. **(B)** shows the distribution of semen leukocyte values in the analytic sample. **(C)** shows the local slope of the fitted spline curve across the observed semen leukocyte range. The models were adjusted for age, abstinence days, semen volume, and semen pH. DFI, sperm DNA fragmentation index.

Adjusted nonlinear exposure-response relationships for key biomarkers are shown in **Figures 4** and **5**, while the corresponding analyses for neutrophil count, lymphocyte count, platelet count, NLR, and SII are provided in **Supplementary Figures 1–5**. Across biomarkers, the exposure-response shapes were heterogeneous, suggesting that a single linear approximation would not adequately describe the full set of relationships.

**Figure 4** shows that albumin demonstrated a clear monotonic pattern. The adjusted curve declined across the main density of the albumin distribution, indicating lower predicted DFI at higher albumin levels. The decline was evident throughout the central range where observations were most concentrated. The local slope remained negative across most of this range and approached zero only toward the upper end, consistent with a diminishing gradient rather than a change in direction. These findings support a broadly expressed inverse association between albumin and DFI.

The nonlinear patterns for leukocyte subtype counts and derived inflammatory indices were more modest and are shown in **Supplementary Figures 1–5**. Neutrophil count showed a shallow increase in predicted DFI across the mid-range followed by attenuation at higher values. Lymphocyte count showed a nonmonotonic pattern, with predicted DFI decreasing toward a mid-range nadir and then increasing slightly at higher values. Platelet count showed the weakest curvature, with largely flat predicted DFI across the central exposure range. NLR and SII showed graded patterns characterized by stronger changes at lower values and attenuation toward the upper range.

**Figure 5** shows that semen leukocytes demonstrated a distinct nonlinear profile with clear separation at low exposure levels. The exposure distribution was markedly right-skewed, with a large mass near zero and a long upper tail. The adjusted curve increased rapidly from very low leukocyte values and then approached a plateau as leukocyte levels increased. The local slope was strongly positive near zero and declined quickly toward zero across the mid range, indicating that the association was concentrated within the low leukocyte range containing most examinations. This pattern suggests a nonlinear association between semen leukocytes and DFI, with a steeper rise at lower exposure values and attenuation at higher values, but it should not be interpreted as evidence of a biologic threshold.

Taken together, the spline analyses indicate that the exposure-response shape depends on the biomarker. Albumin showed a consistent monotonic inverse association, whereas semen leukocytes showed a marked saturating pattern. The additional curves presented in the **Supplementary Material** support the presence of more modest or nonmonotonic nonlinear relationships for several inflammatory markers.

### 3.6 Multivariable models prediction performance and robustness checks

**Figure 6:**
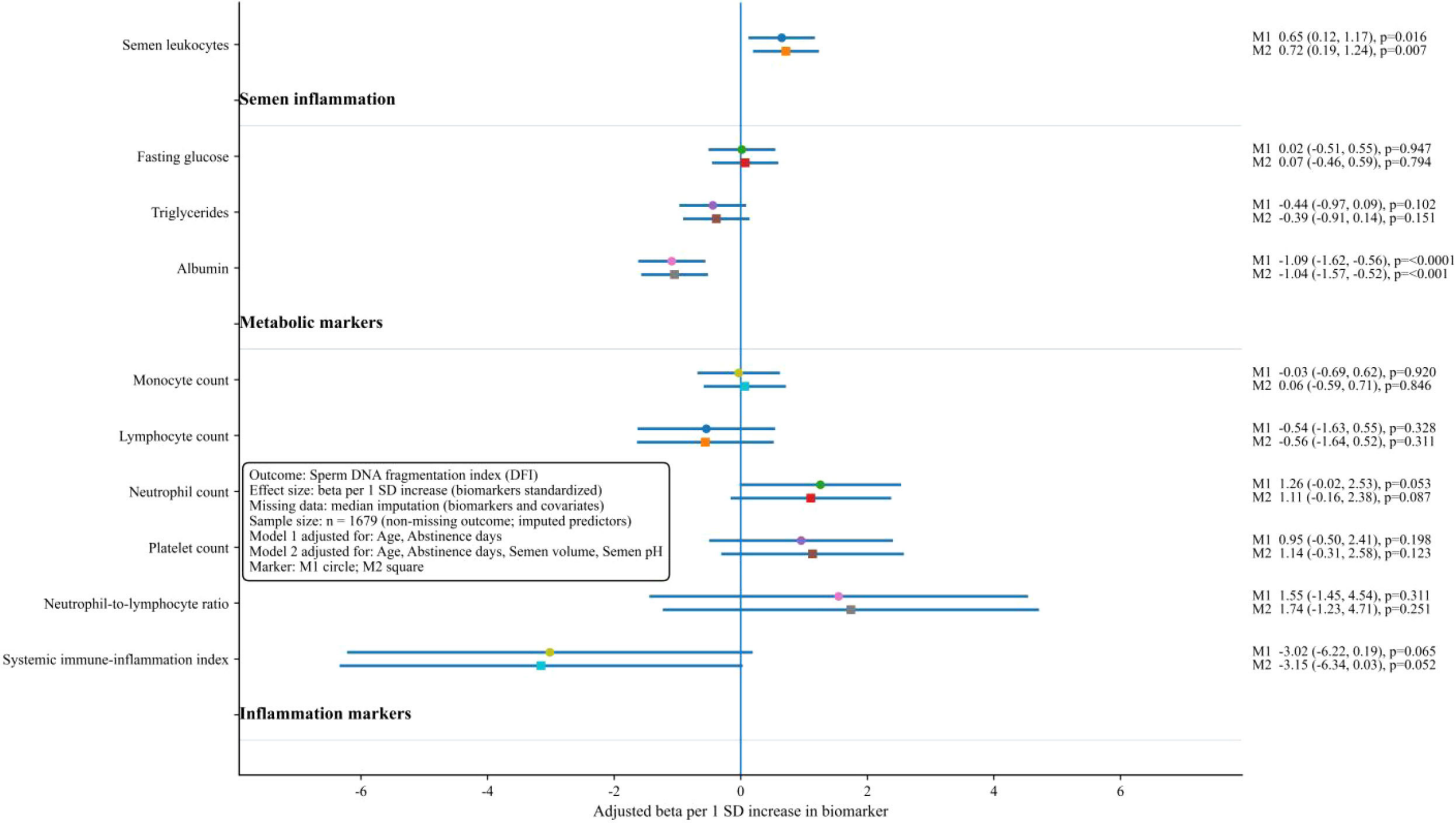
Forest plot of adjusted associations between selected biomarkers and DFI in multivariable linear regression. Effect estimates are shown per 1 standard deviation increase in each biomarker. Points represent regression coefficients and horizontal lines represent 95% confidence intervals. Two nested models are presented: a model adjusted for age and abstinence days, and an expanded model additionally adjusted for semen volume and semen pH. DFI, sperm DNA fragmentation index.

**Figure 7:**
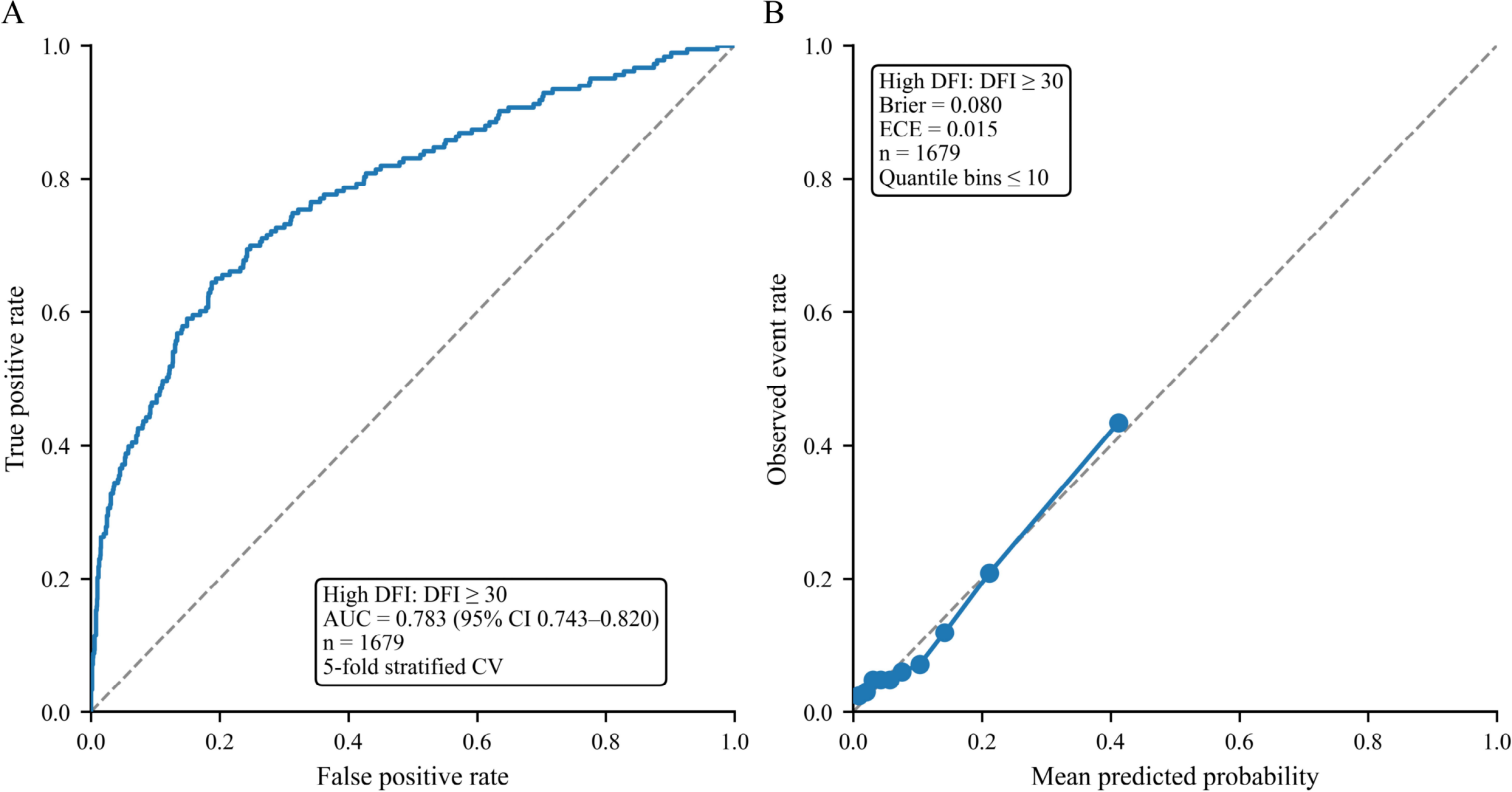
Discrimination and calibration performance of the prediction model for high sperm DNA fragmentation. **(A)** ROC curve for prediction of high sperm DNA fragmentation based on out-of-fold predicted probabilities obtained using 5-fold stratified cross-validation. High DFI was defined as DFI ≥30%. The image reports the AUC and its 95% confidence interval. **(B)** Calibration plot for prediction of high sperm DNA fragmentation. Observed event rates are plotted against mean predicted probabilities across quantile-based risk bins. The diagonal line indicates perfect calibration. Calibration performance is summarized by the Brier score and ECE. DFI, sperm DNA fragmentation index; ROC, receiver operating characteristic; AUC, area under the curve; ECE, expected calibration error.

**Figure 8:**
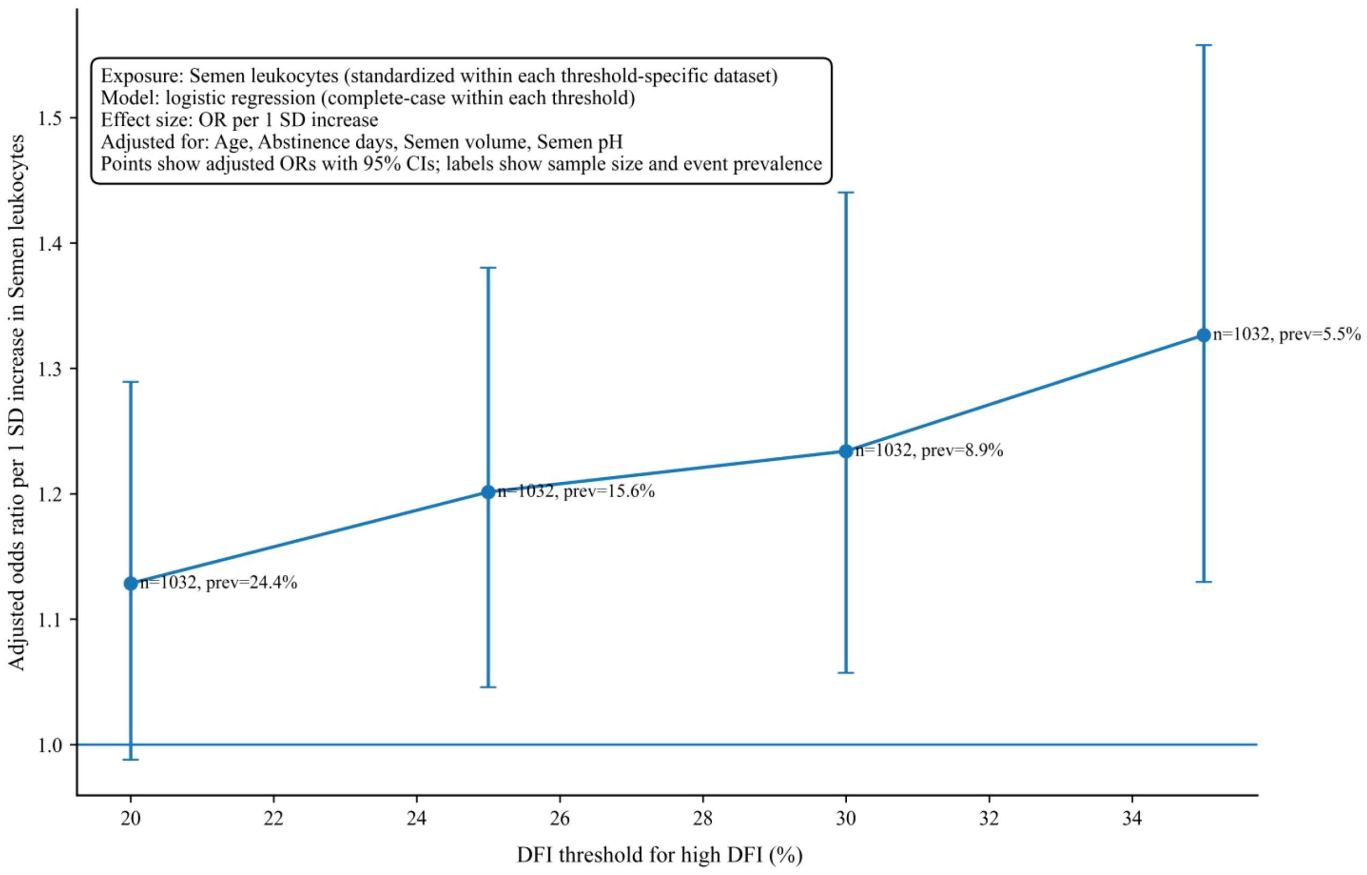
Adjusted odds ratios are shown per 1 standard deviation increase in semen leukocyte level across alternative thresholds used to define high DFI. Horizontal lines indicate 95% confidence intervals, and corresponding event prevalence is also displayed. DFI, sperm DNA fragmentation index.

The results from the multivariable modeling, prediction, and robustness analyses, presented in **Figures 6**–**8**, provide a comprehensive evaluation of the factors influencing DFI. These analyses begin by quantifying the effect estimates of candidate biomarkers on continuous DFI, adjusted for covariates. They then assess the ability of a multivariable predictor set to discriminate examinations with high DFI through out-of-fold validation. Finally, the analyses explore the stability of key associations across different operational definitions of high DFI.

In multivariable linear regression models with DFI as a continuous outcome, adjusted effect estimates are shown in **Figure 6**. Effect sizes are expressed per 1 standard deviation increase in each biomarker, and two nested adjustment models are reported. The more parsimonious model adjusted for age and abstinence days, and the expanded model additionally adjusted for semen volume and semen pH. Across biomarkers, two signals remained robust and stable across adjustment sets. Semen leukocytes showed a positive association with DFI in both models, with the point estimate increasing slightly after additional semen covariate adjustment. Albumin showed a strong inverse association with DFI in both models, with nearly identical estimates after additional adjustment, indicating that the albumin association was not materially altered by inclusion of semen volume and semen pH. Triglycerides showed an inverse tendency, whereas fasting glucose was close to the null, and both had wide confidence intervals spanning the null. For blood cell counts and derived inflammatory indices, point estimates were generally centered near zero, and confidence intervals were wide, suggesting limited evidence for independent linear effects of these markers on DFI after covariate adjustment in the modeled framework. The consistency of the leukocyte and albumin estimates across the two adjustment sets supports their prioritization as key biomarkers in the multivariable setting.

Discrimination performance for prediction of high DFI is shown in **Figure 7**. Using the main definition of high DFI as DFI at least 30, the logistic regression model evaluated under 5 fold stratified cross validation achieved an area under the ROC curve of 0.783 with a 95% confidence interval of 0.743 to 0.820. The ROC curve rose above the diagonal reference line across much of the false positive rate range, indicating discrimination better than chance for examinations with and without high DFI in internal validation. The annotation indicates that the model used 15 predictors and was evaluated out of fold, reflecting expected performance for new observations drawn from the same setting.

Calibration performance is summarized in **Figure 7**. The calibration curve compared observed event rates with mean predicted probabilities across quantile based risk bins. The curve tracked closely with the diagonal reference line over the lower to mid predicted probability range, where most observations were concentrated, indicating good agreement between predicted and observed risk in the region most relevant to typical clinical decision thresholds. Quantitative metrics were consistent with this visual impression. The Brier score was 0.080, indicating low average squared prediction error, and the ECE was 0.015, indicating small average deviation between predicted and observed probabilities across bins. The highest risk bin showed greater deviation from the diagonal, which is consistent with fewer observations at the extreme right tail of predicted risk and wider bin level uncertainty, but the overall pattern suggests reasonable calibration of the model within the observed probability range in this internally validated dataset.

Robustness of the principal association under alternative definitions of high DFI is shown in **Figure 8**. This analysis focused on semen leukocytes and estimated adjusted odds ratios per 1 standard deviation increase under multiple DFI thresholds. The direction of association remained positive across all evaluated thresholds, and point estimates increased modestly as the threshold increased. This pattern indicates that the leukocyte association was not dependent on a single cutoff definition. The figure also reports event prevalence at each threshold, which declined at higher thresholds as expected, yet the association estimate remained stable in direction and magnitude, supporting consistency across clinically plausible high DFI definitions. Taken together with the linear regression results, these findings suggest that semen leukocytes represent a robust marker associated with both continuous DFI and elevated DFI risk.

In summary, multivariable modeling identified two biomarkers with consistent adjusted associations, semen leukocytes with higher DFI and albumin with lower DFI, with effect estimates that were stable across nested covariate adjustment sets. The multivariable logistic model demonstrated good out of fold discrimination and favorable calibration, and the key association with semen leukocytes remained consistent under alternative high DFI thresholds, supporting robustness of the main inference.

## 4 Discussion

### 4.1 Main findings and interpretation

In this retrospective observational study based on routinely collected clinical data from the Reproductive Center of Huzhou Maternity and Child Health Care Hospital, DFI showed a right-skewed distribution and a non-trivial prevalence of high DNA fragmentation under the prespecified threshold. DFI increased across age strata, and the prevalence of high DFI rose markedly in older groups. Across abstinence duration groups, differences in DFI were comparatively modest, suggesting that abstinence duration explained only a limited proportion of variation in DFI at the population level.

We then characterized how DFI related to biomarkers across systemic, conventional semen, and CASA kinematic domains. The systemic inflammatory and metabolic panel exhibited limited pairwise correlations with DFI, with albumin emerging as the most prominent systemic correlate and showing an inverse relationship with DFI. Conventional semen parameters and CASA kinematics showed clearer and more coherent associations. Higher DFI was associated with lower progressive motility, lower total motility, lower normal morphology, and reduced CASA motion measures. Semen leukocytes correlated positively with DFI and ranked among the strongest semen-domain correlates.

Spline-based analyses further indicated that the exposure-response shapes were heterogeneous rather than uniformly linear. Albumin showed a broadly monotonic inverse association with DFI across the main exposure range, whereas semen leukocytes showed a steep rise at low values followed by attenuation, consistent with a saturating pattern. Several leukocyte-related measures and derived inflammatory indices showed more modest or non-monotonic changes. In multivariable linear regression, semen leukocytes and albumin retained stable associations with DFI across nested adjustment sets. In prediction analyses, a multivariable logistic model for high DFI achieved good discrimination and favorable calibration in out-of-fold evaluation, and the association between semen leukocytes and high DFI remained directionally consistent under alternative DFI thresholds.

### 4.2 Biological interpretation and relation to previous work

The most consistent semen-domain signal in the present study was the positive association between semen leukocytes and DFI. Semen leukocytes may reflect an inflammatory microenvironment within the male reproductive tract that is compatible with oxidative stress and impaired sperm DNA integrity. This interpretation is consistent with prior reviews and clinical studies showing that leukocytospermia is linked to reactive oxygen species generation, impaired sperm function, and higher sperm DNA damage (19–23, 31–33). In this context, the nonlinear pattern observed here should be interpreted cautiously. Predicted DFI increased more steeply at lower semen leukocyte values and then attenuated at higher values. This pattern suggests a nonlinear association concentrated in the lower exposure range, but the observational design does not permit inference regarding an underlying biologic threshold or a specific mechanistic transition.

Albumin showed a consistent inverse association with DFI and remained stable after adjustment for semen covariates. Although albumin is not a semen-specific biomarker, it is commonly interpreted as a marker of systemic inflammatory and nutritional status and may also reflect broader antioxidant buffering capacity. From a mechanistic perspective, lower albumin could therefore be compatible with a biologic milieu that is more vulnerable to oxidative stress, a pathway that has been repeatedly implicated in sperm dysfunction and DNA damage (15–18). We therefore interpret the albumin signal cautiously as a plausible systemic correlate of sperm DNA integrity rather than as evidence of a direct causal pathway.

The inverse associations of DFI with motility and CASA kinematic parameters were also biologically coherent. Oxidative stress and sperm DNA damage do not occur in isolation but are often accompanied by broader defects in sperm structure and function. Previous studies and recent reviews have reported that elevated DFI is commonly associated with poorer semen quality, including lower motility and less favorable morphology, although the magnitude of these relationships varies across populations and laboratory methods (32, 33, 35, 36). In our dataset, CASA velocity descriptors and related measures were tightly correlated with one another and were concordantly inversely associated with DFI, supporting the interpretation that sperm DNA integrity and motion phenotype represent complementary dimensions of sperm quality.

The observed increase in DFI with age in our cohort is also consistent with recent literature showing that advancing male age is associated with deterioration in sperm DNA integrity and broader sperm quality measures (29, 30, 34). Likewise, the inverse relationships with motility and morphology observed in the present study align with recent data indicating that lower motility rates and abnormal morphology are associated with higher levels of sperm DNA fragmentation (28, 29, 35, 36). Taken together, these comparisons suggest that our findings are not isolated observations but fit within a growing body of evidence that places sperm DNA integrity at the intersection of age-related change, inflammatory stress, and impaired semen function.

### 4.3 Clinical relevance and study appraisal

A distinctive contribution of the present study is the joint evaluation of systemic biomarkers, conventional semen parameters, CASA kinematics, and semen leukocytes within a single routinely collected clinical dataset. Rather than examining one biomarker domain in isolation, we used a common analytic framework combining correlation analysis, flexible exposure-response modeling, multivariable effect estimation, and internally validated prediction. Within this framework, semen leukocytes emerged as the most robust semen-related inflammatory marker, whereas albumin emerged as the clearest systemic correlate of DFI. This integrated perspective may be particularly useful in real-world reproductive medicine, where DFI testing is often selective but routine semen and blood measurements are more broadly available.

These findings may have potential relevance for settings in which DFI testing is available but not routinely performed for all patients. Semen leukocytes and albumin are commonly measured in routine care, and their consistent associations with DFI suggest that they may warrant further study as complementary markers in the evaluation of sperm DNA integrity. More broadly, recent reviews have emphasized that sperm DNA fragmentation testing is most informative when interpreted alongside routine semen analysis and clinical context, rather than as a stand-alone metric (11, 12, 32, 33, 36). The internally validated prediction model in our study showed good discrimination and favorable calibration; however, these results should be interpreted as exploratory model development findings, and external validation is required before any clinical application.

This study has several strengths. It was based on a relatively large real-world clinical dataset of consecutive semen examinations with available DFI assessment. It also integrated multiple biomarker domains and moved beyond simple bivariate analysis by combining correlation summaries, nonlinear spline modeling, multivariable effect estimation, internal cross-validated prediction, and threshold-robustness assessment. The convergence of results across these analytic layers strengthens the overall internal coherence of the study.

The study also has limitations. Because this was a retrospective observational analysis of routine clinical records, unmeasured confounding cannot be fully excluded. In particular, some potentially relevant lifestyle covariates, including body mass index and smoking status, were not consistently available for inclusion in the adjusted analyses. In addition, because the analytic unit was the semen examination record, some individuals may have contributed more than one examination during the study period; accordingly, the present study was designed to characterize examination-record level association patterns in routine clinical data rather than within-individual longitudinal change. Biomarker completeness varied across domains because laboratory tests were not uniformly obtained in routine care, resulting in analysis-specific effective sample sizes; therefore, findings for systemic biomarkers should be interpreted cautiously. In addition, this was a single-center study, and external validation is needed, especially for the predictive model. Finally, the present analyses were designed to characterize association patterns rather than establish causality, and the biological interpretations should be regarded as clinically plausible but not definitive.

## 5 Conclusion

In this retrospective clinical study from a reproductive center, DFI increased with age and was inversely related to semen motility and CASA kinematics. Semen leukocytes and albumin emerged as the most consistent biomarkers associated with sperm DNA fragmentation, with evidence of nonlinear exposure-response patterns. An internally validated multivariable model showed good discrimination and calibration for identifying high DFI within this dataset, and key associations were consistent across alternative high-DFI thresholds. These findings support further investigation of routinely available semen and blood biomarkers in relation to sperm DNA integrity, while external validation is required before broader clinical application.

## Data Availability

The raw data supporting the conclusions of this article will be made available by the authors, without undue reservation.
