# Supplementary material for: Association of semen leukocytes with sperm DNA fragmentation in a clinical cohort
Source: Front Endocrinol (Lausanne). 2026 May 21;17:1832477. doi: 10.3389/fendo.2026.1832477 (PMC13233189; doi:10.3389/fendo.2026.1832477)
Supplement: Supplementary file 1 [file DataSheet1.docx]

| 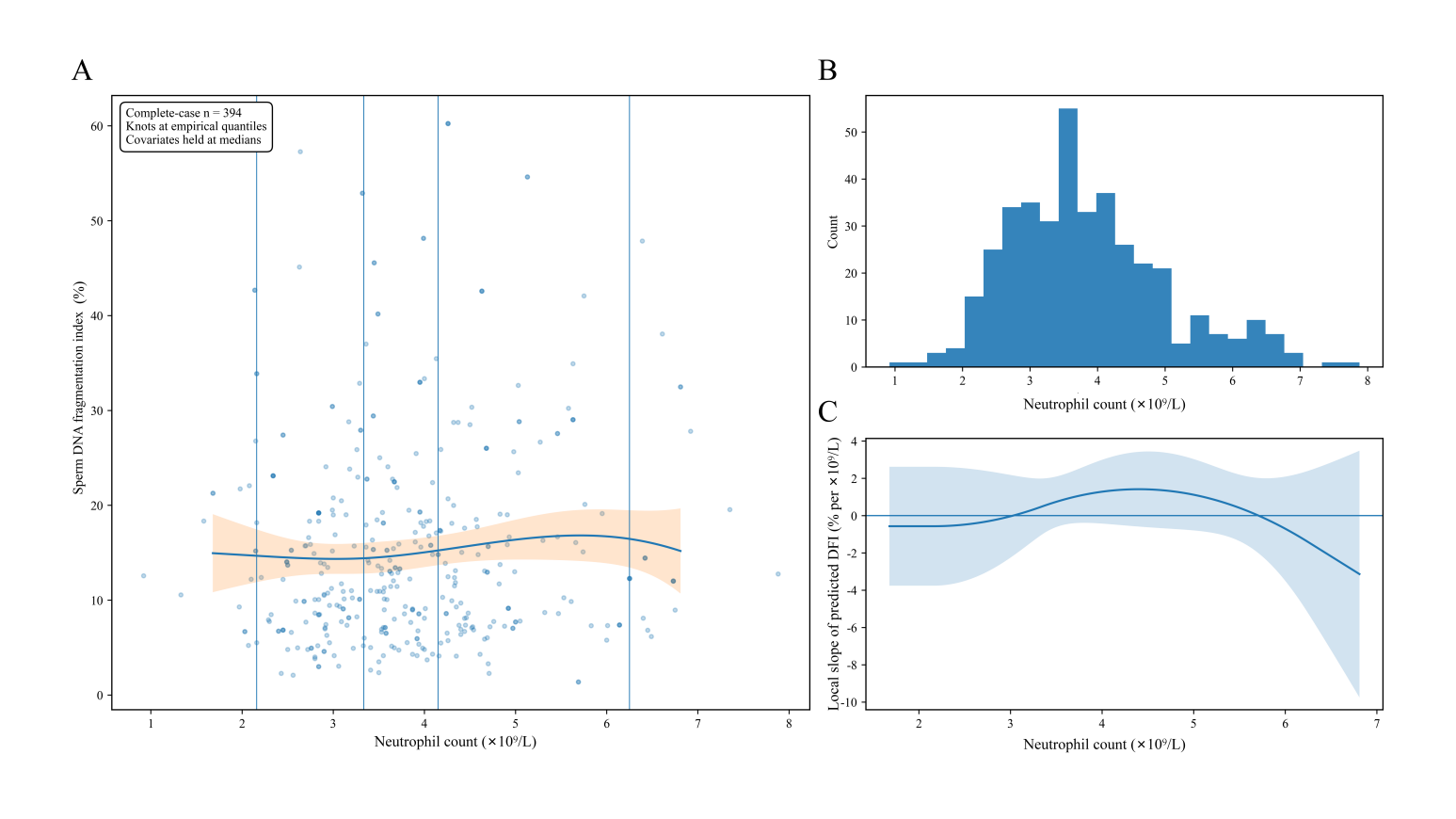 |
| --- |
| Supplementary Fig.S1. Natural cubic spline models showing the nonlinear association between neutrophil count and sperm DNA fragmentation index. The y-axis in panel A represents the predicted mean sperm DNA fragmentation index (DFI) and its 95% confidence interval. The x-axis shows neutrophil count. (A) Adjusted spline curve for the association between neutrophil count and DFI. (B) Distribution of neutrophil count in the analytic sample. (C) Local slope of the fitted spline curve across the observed neutrophil count range. The models were adjusted for age, abstinence days, semen volume, and semen pH. DFI, sperm DNA fragmentation index. |

| 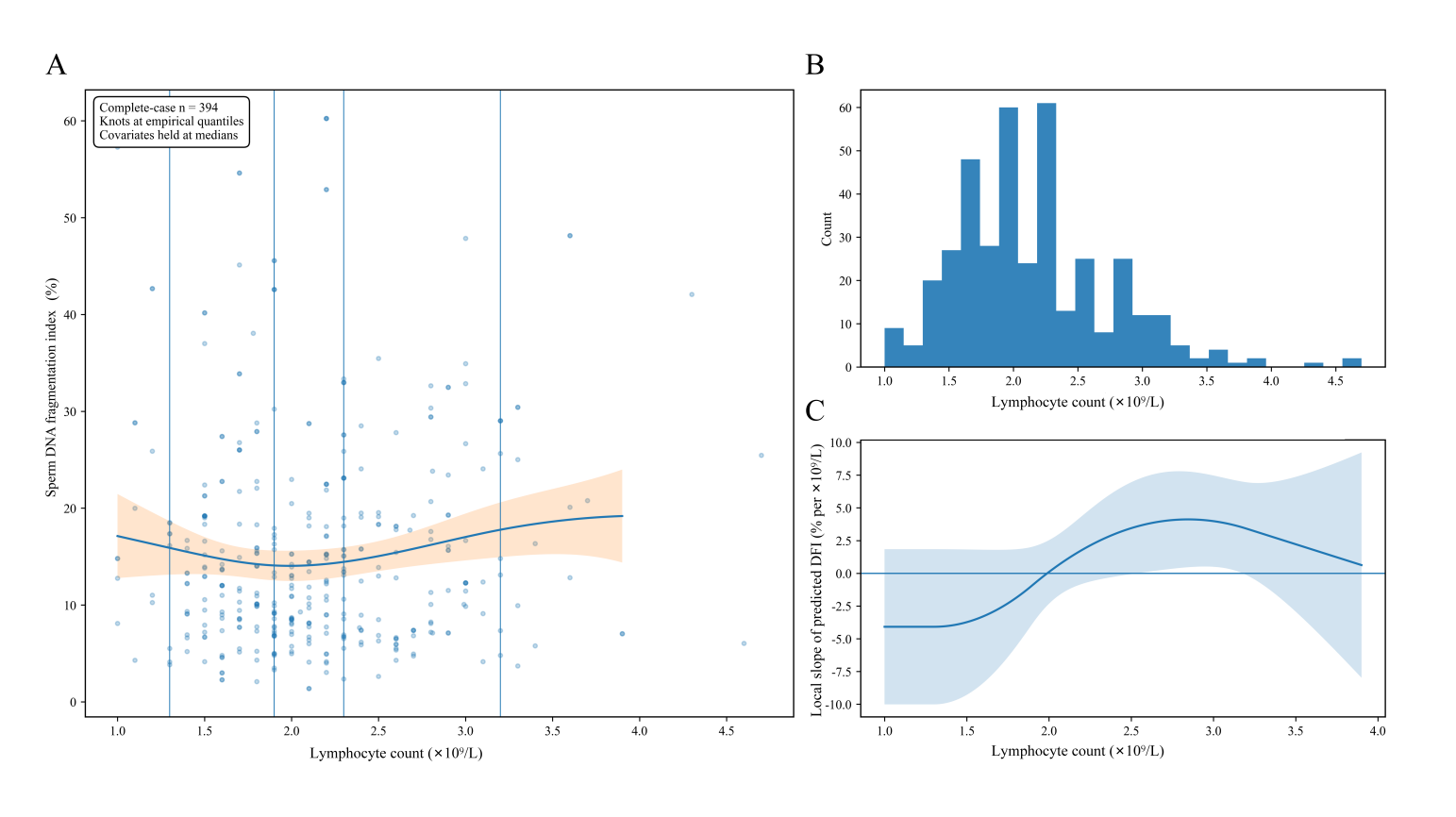 |
| --- |
| Supplementary Fig.S2. Natural cubic spline models showing the nonlinear association between lymphocyte count and sperm DNA fragmentation index. The y-axis in panel A represents the predicted mean sperm DNA fragmentation index (DFI) and its 95% confidence interval. The x-axis shows lymphocyte count. (A) Adjusted spline curve for the association between lymphocyte count and DFI. (B) Distribution of lymphocyte count in the analytic sample. (C) Local slope of the fitted spline curve across the observed lymphocyte count range. The models were adjusted for age, abstinence days, semen volume, and semen pH. DFI, sperm DNA fragmentation index. |

| 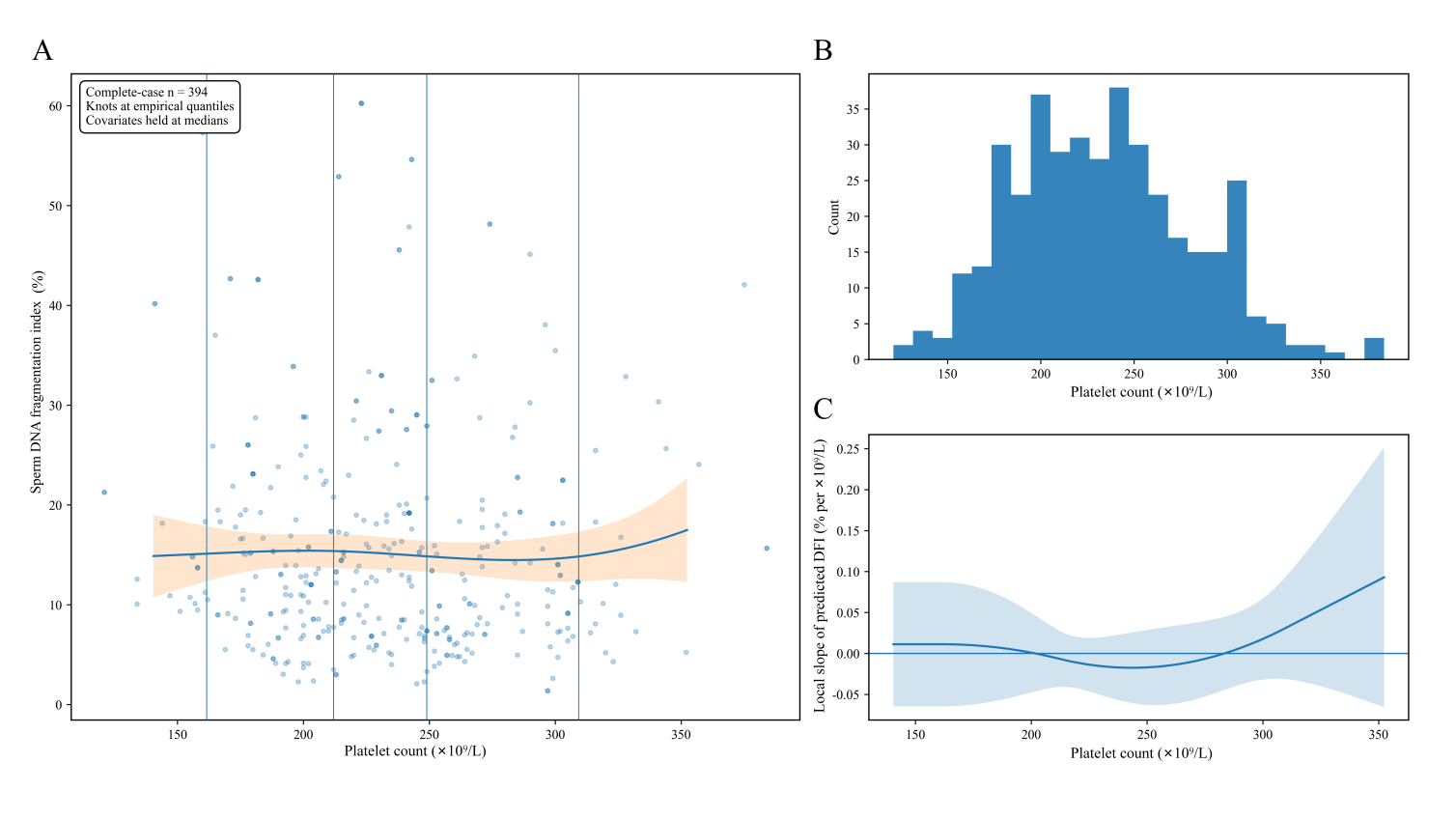 |
| --- |
| Supplementary Fig.S3. Natural cubic spline models showing the nonlinear association between platelet count and sperm DNA fragmentation index. The y-axis in panel A represents the predicted mean sperm DNA fragmentation index (DFI) and its 95% confidence interval. The x-axis shows platelet count. (A) Adjusted spline curve for the association between platelet count and DFI. (B) Distribution of platelet count in the analytic sample. (C) Local slope of the fitted spline curve across the observed platelet count range. The models were adjusted for age, abstinence days, semen volume, and semen pH. DFI, sperm DNA fragmentation index. |

| 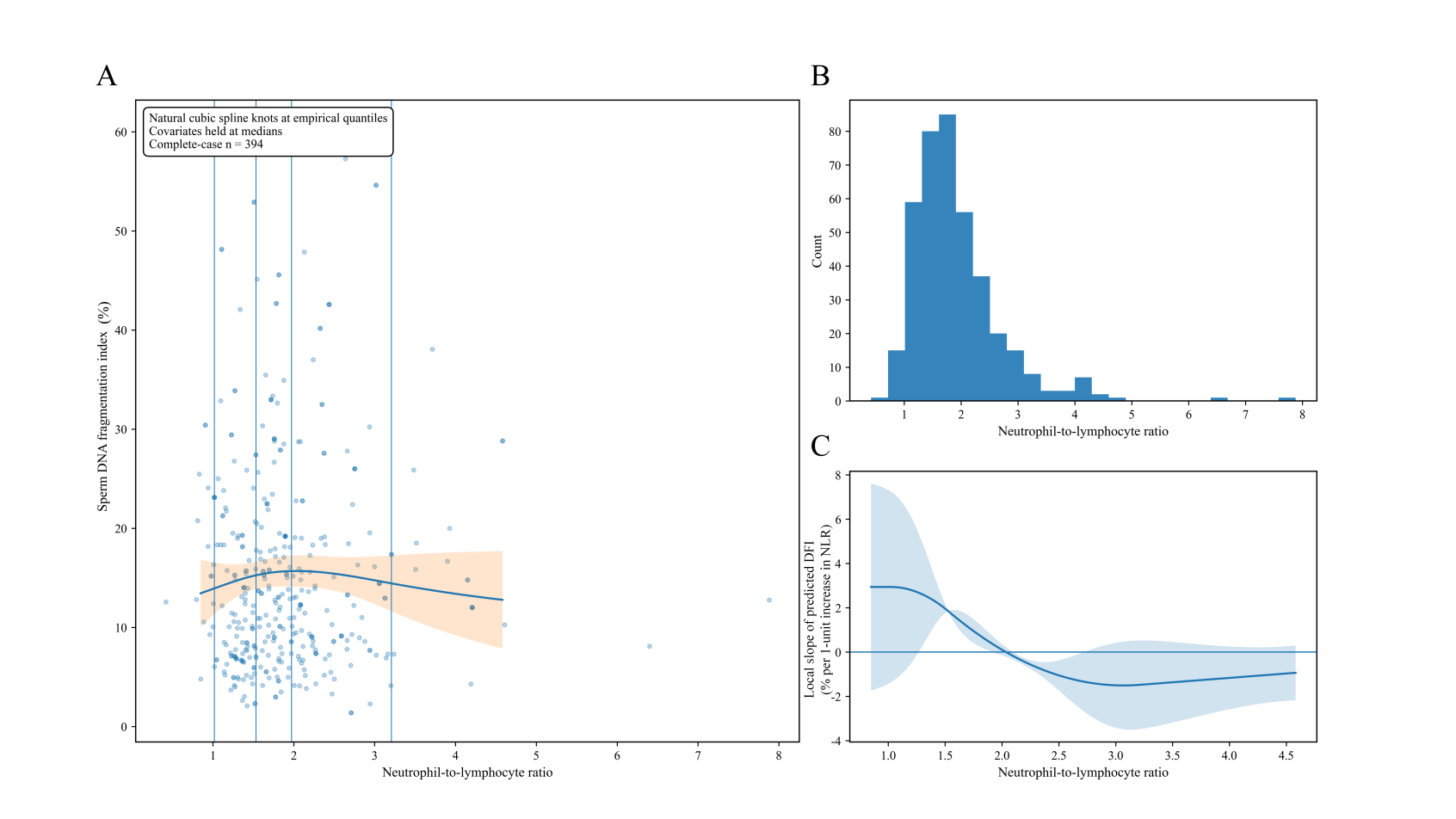 |
| --- |
| Supplementary Fig.S4. Natural cubic spline models showing the nonlinear association between NLR and sperm DNA fragmentation index. The y-axis in panel A represents the predicted mean sperm DNA fragmentation index (DFI) and its 95% confidence interval. The x-axis shows neutrophil-to-lymphocyte ratio (NLR). (A) Adjusted spline curve for the association between NLR and DFI. (B) Distribution of NLR values in the analytic sample. (C) Local slope of the fitted spline curve across the observed NLR range. The models were adjusted for age, abstinence days, semen volume, and semen pH. DFI, sperm DNA fragmentation index; NLR, neutrophil-to-lymphocyte ratio. |

| 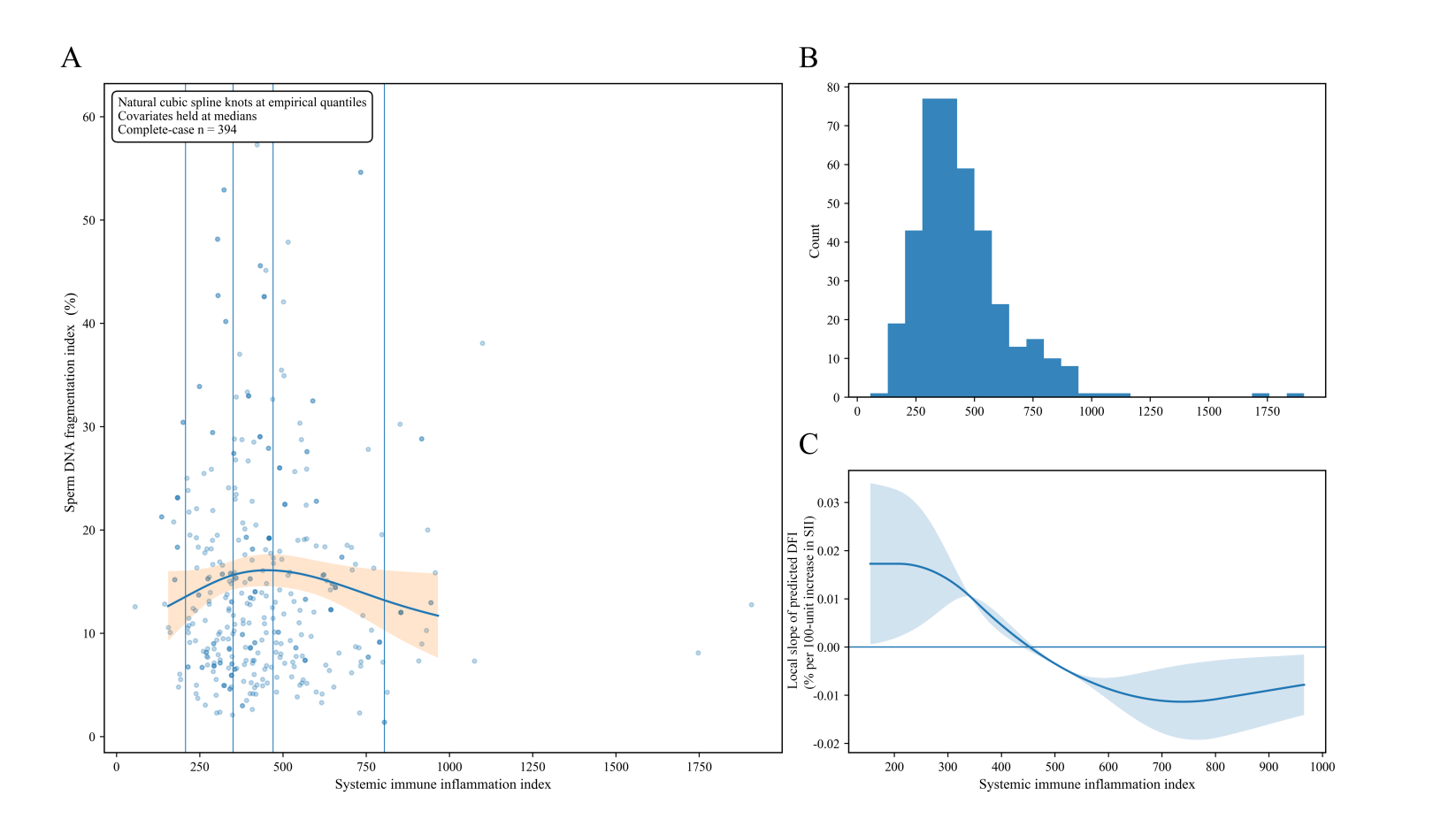 |
| --- |
| Supplementary Fig. S5. Natural cubic spline models showing the nonlinear association between SII and sperm DNA fragmentation index. The y-axis in panel A represents the predicted mean sperm DNA fragmentation index (DFI) and its 95% confidence interval. The x-axis shows systemic immune-inflammation index (SII). (A) Adjusted spline curve for the association between SII and DFI. (B) Distribution of SII values in the analytic sample. (C) Local slope of the fitted spline curve across the observed SII range. The models were adjusted for age, abstinence days, semen volume, and semen pH. DFI, sperm DNA fragmentation index; SII, systemic immune-inflammation index. |
